# Comparison of the performance of three PCR assays for the detection and differentiation of *Theileria orientalis* genotypes

**DOI:** 10.1186/s13071-015-0812-7

**Published:** 2015-03-31

**Authors:** Piyumali K Perera, Robin B Gasser, David J Pulford, Mark A Stevenson, Simon M Firestone, Andrew M J McFadden, Abdul Jabbar

**Affiliations:** Faculty of Veterinary and Agricultural Sciences, The University of Melbourne, Werribee, Victoria 3030 Australia; Investigation and Diagnostic Centres and Response, Ministry for Primary Industries, Wellington, New Zealand

**Keywords:** *Theileria orientalis*, Multiplexed tandem PCR, PCR-high resolution melting analysis, TaqMan qPCR assay, Anaemia, *Ikeda*

## Abstract

**Background:**

Oriental theileriosis is a tick-borne disease of bovines caused by the members of the *Theileria orientalis* complex. Recently, we developed a multiplexed tandem (MT) PCR to detect, differentiate and quantitate four genotypes (i.e., *buffeli*, *chitose*, *ikeda* and *type* 5) of *T. orientalis*. In this study, we used MT PCR to assess the prevalence and infection intensity of four *T. orientalis* genotypes in selected cattle herds that experienced oriental theileriosis outbreaks in New Zealand, and compared the sensitivities and specificities of MT PCR, PCR-high resolution melting (PCR-HRM) and a TaqMan® qPCR.

**Methods:**

MT PCR, PCR-HRM analysis for *T. orientalis* and a TaqMan® qPCR assay for *ikeda* genotype were employed to test 154 and 88 cattle blood samples from North (where oriental theileriosis outbreaks had occurred; designated as Group 1) and South (where no outbreaks had been reported; Group 2) Islands of New Zealand, respectively. Quantitative data from MT PCR assay were analyzed using generalized linear model and paired-sample *t*-test. The diagnostic specificity and sensitivity of the assays were estimated using a Bayesian latent class modeling approach.

**Results:**

In Group 1, 99.4% (153/154) of cattle were test-positive for *T. orientalis* in both the MT PCR and PCR-HRM assays. The apparent prevalences of genotype *ikeda* in Group 1 were 87.6% (134/153) and 87.7% (135/154) using the MT PCR and Ikeda TaqMan® qPCR assays, respectively. Using the MT PCR test, all four genotypes of *T. orientalis* were detected. The infection intensity estimated for genotype *ikeda* was significantly higher (*P* = 0.009) in severely anaemic cattle than in those without anaemia, and this intensity was significantly higher than that of *buffeli* (*P* < 0.001) in the former cattle. Bayesian latent class analysis showed that the diagnostic sensitivities (97.1-98.9%) and specificities (96.5-98.9%) of the three PCR assays were very comparable.

**Conclusion:**

The present findings show the advantages of using the MT PCR assay as a useful tool for in-depth epidemiological and transmission studies of *T. orientalis* worldwide.

## Background

*Theileria* species (Apicomplexa: Piroplasmida; Theileriidae) are tick-transmitted intracellular protists that infect various domestic and wild ruminants worldwide [1]. Depending on their pathogenicity, *Theileria* species can be divided into two groups: (i) those that transform host cells (including *T. annulata* and *T. parva*) and (ii) those that do not (e.g., *Theileria orientalis* complex) [2]. *Theileria annulata* and *T. parva* cause the most severe forms of bovine theileriosis, whereas members of *T. orientalis* complex were recognized to usually cause a less severe form (oriental theileriosis) in cattle. However, in recent years, *T. orientalis* has been associated with significant outbreaks of oriental theileriosis in Australia [3,4] and New Zealand [5,6], causing pyrexia, haemolytic anaemia, productivity losses, abortions and/or mortality in dairy and beef cattle [3-8].

Using the sequence of the major piroplasm surface protein (*MPSP*) gene, to date, at least 11 *T. orientalis* genotypes (designated *chitose* or *type* 1, *ikeda* or *type* 2, *buffeli* or *type* 3, *types* 4 to 8, and *N1* to *N3*) have been identified globally [9]. Genotypes *chitose* and *ikeda* are proposed to be associated with severe disease in cattle in the Asia-Pacific region [3-7,10-12].

Recently, McFadden et al. [5] reported that genotype *chitose* was linked to an outbreak of oriental theileriosis in apparently naïve cattle being transported to the northern part of the North Island of New Zealand. Since August 2012, the number of theileriosis outbreaks has increased considerably in this geographical region, and in some herds, there have been significant morbidity, mortality and productivity losses [6]. In recent studies, the identification of genotypes of *T. orientalis* was carried out on samples collected from herds with theileriosis outbreaks, and genotype *ikeda* was identified [6,13].

Clinical signs, haematology, serology and/or molecular tools have been used for the diagnosis of theileriosis in New Zealand [5,13]; however, owing to increasing numbers of oriental theileriosis outbreaks in this country, there has been a need for a rapid and accurate method of identification of pathogenic genotypes of *T. orientalis*. Therefore, Pulford et al. (D. J. Pulford, and A. M. J. McFadden, unpublished observations) developed PCR-high resolution melting (PCR-HRM) and TaqMan® qPCR methods employing the *MPSP* gene as a marker for the detection of genotype *ikeda* of *T. orientalis* in blood samples from cattle in New Zealand. Although the combined use of the PCR-HRM and TaqMan® qPCR allows the identification of the *ikeda* genotype, these assays do not specifically identify or differentiate other genotypes of *T. orientalis* (i.e., *buffeli*, *chitose* and *type* 5), or quantify the amount of DNA of these genotypes (D. J. Pulford, and A. M. J. McFadden, unpublished observations).

To overcome the limitations associated with conventional molecular tools (such as low diagnostic sensitivity and/or specificity, quantitation and time required for testing), Perera et al. [14] recently established and validated a multiplexed tandem PCR (MT PCR) assay for the simultaneous detection, differentiation and quantitation of four of the commonest genotypes (i.e., *buffeli*, *chitose*, *ikeda* and *type* 5) of *T. orientalis* in Australasia*.* Subsequently, Perera et al. [15] used MT PCR to estimate the prevalence and intensity of these four genotypes in cattle in 15 dairy herds in the State of Victoria, Australia, and demonstrated the utility, high performance, throughput and convenience of the assay for diagnostic and epidemiological applications. Using MT PCR, these authors were able to determine the prevalence of four common *T. orientalis* genotypes (i.e., *buffeli*, *chitose*, *ikeda* and *type* 5) and quantify the DNA copy number for each of these genotypes in individual cattle.

As three novel molecular-diagnostic assays have been developed independently in Australia (MT PCR) and New Zealand (PCR-HRM and TaqMan® qPCR), the aims of the present study were to: (i) employ MT PCR to estimate the prevalence and infection intensity of *T. orientalis* genotypes *buffeli*, *chitose*, *ikeda* and *type* 5 in selected cattle herds that had experienced oriental theileriosis outbreaks in New Zealand, and (ii) compare the sensitivities and specificities of all three assays.

## Methods

### Farms, demographic characteristics of cattle, and blood collection

**Figure 1:**
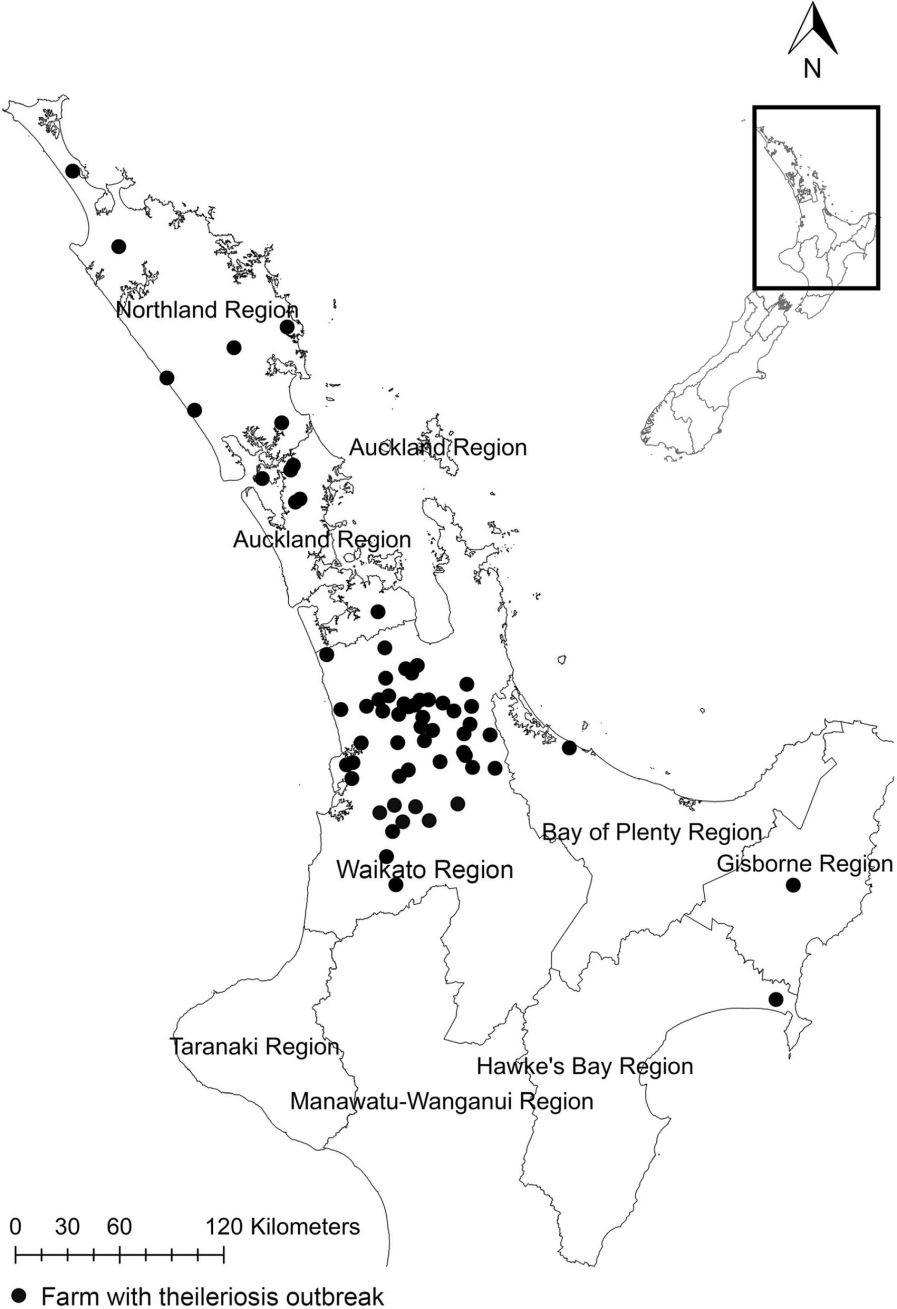
**Map showing locations of the recent oriental theileriosis outbreaks occurred in North Island, New Zealand.**

Blood samples (*n* = 154) were collected by registered veterinarians from 103 beef (Angus, Hereford or crosses) or dairy (Friesian or crosses) herds experiencing oriental theileriosis (Group 1) in four regions (i.e., Auckland, East Cape, Northland and Waikato) from the North Island, New Zealand, between July and December 2013 (Figure 1; Table 1). The impact of this disease was recorded as ‘moderate’ (1-2 cattle died) on 61 farms and ‘severe’ (≥5 cattle died and > 30% of the herd were anaemic) on one farm (Figure 1). Clinical signs recorded were acute death, pale vulval mucous membranes, anorexia, lethargy, tachycardia and/or tachypnoea. At necropsy, affected cattle were often jaundiced, with watery un-clotted blood in major blood vessels. Information on age and breed of cattle in each herd (where possible) was obtained. In addition to samples from cattle in herds suffering from outbreaks, blood samples (*n* = 88) from nine unaffected herds were randomly collected from asymptomatic beef cattle (Group 2) in the Southland region of the South Island, New Zealand, with no history of theileriosis (Table 1). Although ticks are abundant in North Island, there was no evidence of ticks present in the herds representing Group 2.

**Table 1:** **Apparent prevalence and infection intensity with genotypes of** ***Theileria orientalis*** **complex in New Zealand outbreaks**

**Region**	**No. of farms**	**Farm types (** ***n*** **)** ^**a**^	**Disease groups** ^**b**^	**No. of Samples tested**	**Test-positive by Ikeda TaqMan® qPCR % (proportion)**	**Test-positive by PCR-HRM % (proportion)**	**Test-positive by MT PCR % (proportion)**	**Apparent prevalence of genotype % (proportion)**				**Mean intensity of infection by genotype (mean no. of DNA copies)**			
								***ikeda***	***chitose***	***buffeli***	***type*** **5**	***ikeda***	***chitose***	***buffeli***	***type*** **5**
**North Island**															
Auckland	13	Beef (6)/Dairy (4)	NR/ MO	17	94.1 (16/17)	100 (17/17)	100 (17/17)	94.1 (16/17)	70.6 (12/17)	100 (17/17)	29.4 (5/17)	381,134	15,466	209,763	10,939
East Cape	8	Beef (7)/Dairy (1)	NR/MO	18	44.4 (8/18)	100 (18/18)	100 (18/18)	44.4 (8/18)	77.8 (14/18)	83.3 (15/18)	22.2 (4/18)	64,727	27,164	68,263	9
Northland	14	Beef (5)/Dairy (9)	NR/MO/ANT	32	100 (32/32)	100 (32/32)	100 (32/32)	100 (32/32)	87.5 (28/32)	100 (32/32)	53.1 (17/32)	237,979	129,868	159,724	15,823
Waikato	62	Beef (5)/Dairy (56)	NR/MO/ANT/SO	87	90.8 (79/87)	98.9 (86/87)	98.9 (86/87)	90.7 (78/86)	31.4 (27/86)	100 (86/86)	3.5 (3/86)	420,479	57,012	186,854	859
**Total**	97	Beef (23)/Dairy (70)		154	87.7 (135/154)	99.4 (153/154)	99.4 (153/154)	87.6 (134/153)	52.9 (81/153)	98.0 (150/153)	19.0 (29/153)	349,271	70,684	170,786	11,252
**South Island**															
Southland	9	Beef (9)	NR	88	0% (0/88)	2.3% (2/88)	2.3% (2/88)	0	100 (2/2)	100 (2/2)	100 (2/2)	0	6,916	7,694	22

^a^ *n* denotes the number of farms sampled. For some farms the farm type (dairy or beef) information were not collected.

^b^Disease groups were categorized according to clinical signs and haematological parameters. ‘NR’, ‘ANT’, ‘MO’ and ‘SO’ denote no disease reported, anaemia negative *Theileria* (i.e., anaemia not observed but *Theileria* piroplasms were observed on blood smears), moderate outbreak (i.e., more than one animal with anaemia) and severe outbreak (i.e., with multiple deaths), respectively.

### Haematological examination

The haematocrit (HCT) was determined for each blood sample using an automated haematology analyzer (Cell-Dyn 3700; Abbott Diagnostics Division, Illinois, USA). Based on HCT values, cattle were classified as normal (non-anaemic; > 0.24), mildly anaemic (0.15-0.24), and severely anaemic (<0.15).

### Isolation of genomic DNA

Individual blood samples (i.e., 40 μl of blood from each individual) were diluted 1:5 in sterile Elix water, and genomic DNA was then extracted from 200 μl of each sample using the QIAxtractor system and the DX universal liquid sample DNA extraction kit (Cat. No. 950107; Qiagen) using the manufacturer’s protocol (eluting into 200 μl).

### MT PCR

This assay was conducted using primer pairs (Cat. No. 38170R; AusDiagnostics Pty Ltd, Australia) to the piroplasm surface protein (*p23*) gene (genotype *buffeli*), *MPSP* gene (*chitose*), the first internal transcribed spacer (ITS-1) of nuclear ribosomal DNA (*ikeda*) and again the *MPSP* gene (*type* 5) in the Easy-Plex platform (AusDiagnostics), as described previously [14]. Following primary and secondary amplifications, the peak high resolution melting temperature for each amplicon was compared with the pre-determined reference temperatures representing individual genotypes: *buffeli* (83.6 ± 1.5°C), *chitose* (82.1 ± 1.5°C), *ikeda* (87.4 ± 1.5°C) and *type* 5 (81.6 ± 1.5°C) [14]. Randomly selected amplicons for each genotype were subjected to single-strand conformation polymorphism (SSCP) analysis and sequencing [7,16].

### PCR-HRM

To amplify all genotypes of *T. orientalis*, this assay was performed as described previously (D. J. Pulford, and A. M. J. McFadden, unpublished observations). Briefly, a region of the *MPSP* gene (274 bp) was amplified from genomic DNA (3 μl) using the primers Th.oriMPSP-F (5′-TCCTTGTTTGCCTCGCTCTGCT-3′) and Th.oriMPSP-R (5′-AGGCAGGTCTTTTTGCCGCTGA-3′).

### TaqMan® qPCR

To detect genotype *ikeda* in samples that tested positive by PCR-HRM, the Ikeda TaqMan® qPCR was used as previously described (D. J. Pulford, and A. M. J. McFadden, unpublished observations). A region of the *MPSP* gene of the genotype *ikeda* (86 bp) was amplified from genomic DNA (2 μl) using the primers NZIke1-F (5′-AGTTAACGCCACCGCAGCCG-3′) and the NZIke1-R (5′-ACGCGGTATCCCTCTTCGGCA-3′), using NZIkeda1-Probe ([6FAM]-CGCCTCAAACGCCAACGACG-[BHQ1]) as a specific probe. Following PCR, C*t* values were observed for each sample, and a sample was recorded as ‘test-positive’ if the C*t* value was <38 (D. J. Pulford, and A. M. J. McFadden, unpublished observations).

### Statistical analyses

For MT PCR, the DNA copy numbers of individual genotypes of *T. orientalis* recorded in each blood sample was log-transformed. A generalized linear model was then used to compare the relative intensity of infection for each genotype in individual cattle among three categories of anaemia (i.e., severe anaemia, mild anaemia and no anaemia). A paired-sample *t*-test was used to compare the intensity of infection of the genotypes shown to be dominant, based on prevalence within severely, mildly or non-anaemic cattle (Group 1), using the genotype with the lowest mean DNA copy number as the reference category. The software package SPSS Statistics 22 (IBM) was used for statistical analyses; a *P*-value of < 0.05 was considered statistically significant.

The diagnostic specificity and sensitivity of the MT PCR assay were estimated using a Bayesian latent class modeling approach [17,18] as described previously [14]. Briefly, two sets of conditionally dependent tests (i.e., Set 1; MT PCR and TaqMan® qPCR for genotype *ikeda* only, Set 2; MT PCR and PCR-HRM for *T. orientalis*) were carried out on two distinct cattle populations (i.e., Groups 1 and 2) in the absence of a ‘gold standard’ (i.e., reference samples of known disease status). The apparent prevalence was assumed to be distinct for each population and the diagnostic specificity and sensitivity of each test was assumed to be constant across the two populations. To allow for zero-infection prevalences, the prevalence in each population was modelled as a mixture of point mass at zero and a continuous beta distribution, as described by Branscum et al. [17]. Diagnostic specificity and sensitivity of each test was assumed to be constant across the two populations. The three molecular diagnostic tests were assumed to be dependent (conditional on infection status), because they had the same biological basis, i.e., the detection of DNA of *T. orientalis* genotypes. Information about the diagnostic specificity and sensitivity of the MT PCR [14], and Ikeda TaqMan® qPCR and PCR-HRM were modelled using independent and informative beta-distributions elicited from two technical experts [R. B. Gasser and A. M. J. McFadden, respectively] with knowledge of the study populations and diagnostic test performance. The technical experts were not involved in the sample collection and/or testing. Agreement statistics (prevalence-adjusted bias-adjusted Kappa*,* PABAK) [19] were directly calculated as model outputs. Final inferences were presented as the 50%, 2.5% and 97.5% quantiles of the marginal posterior distributions for each of the parameters, corresponding to a posterior median point estimate and a 95% probability interval (95% PI), respectively.

## Results

### Apparent prevalence of *T. orientalis* genotypes and estimation of infection intensity by MT PCR

**Figure 2:**
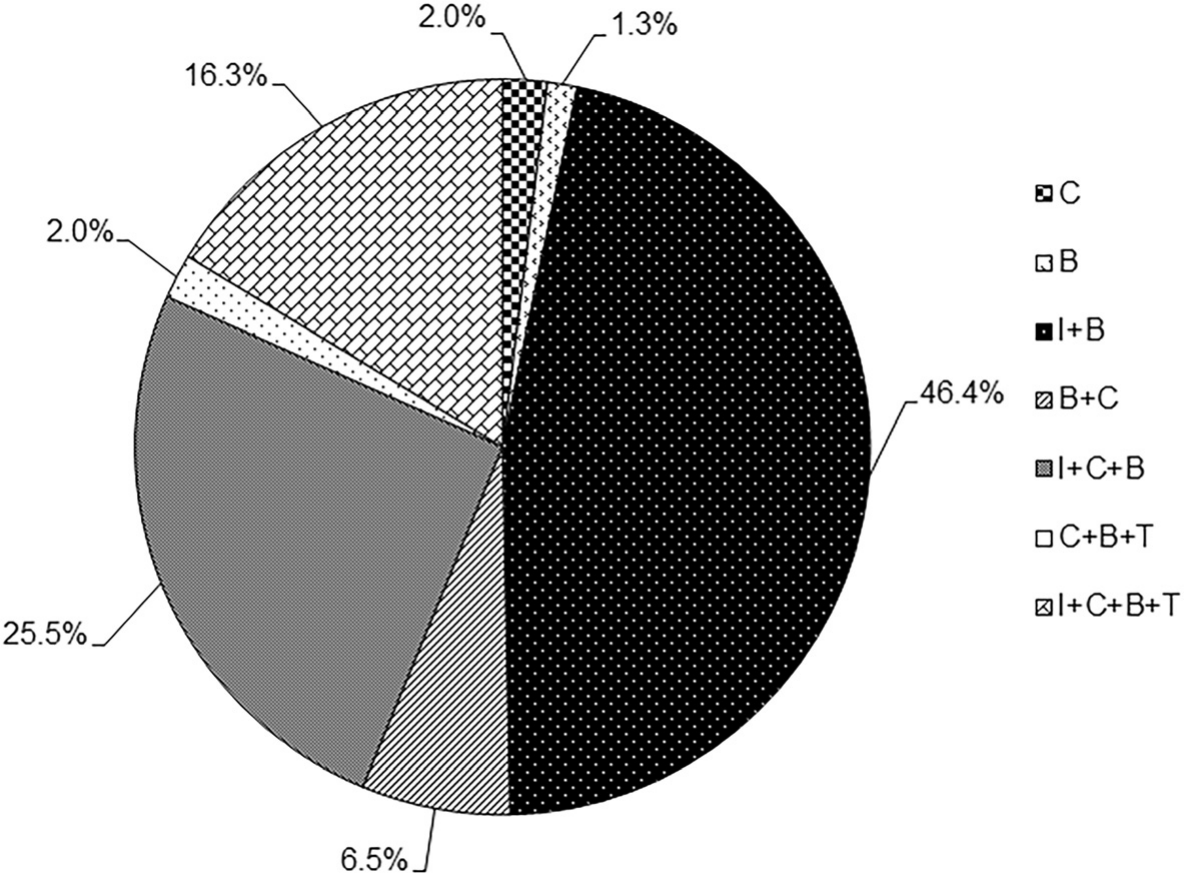
**Prevalence of genotypes of the** ***Theileria orientalis*** **complex detected by the MT PCR assay.** Letters ‘C’, ‘B’, ‘I’ and ‘T’ denote single infections by genotypes *chitose*, *buffeli*, *ikeda* and *type* 5, respectively. Various combinations of letters with the sign ‘+’ denote mixed infections with two or more genotypes.

In Group 1, 99.4% (153/154) of cattle sampled from North Island were test-positive for *T. orientalis* by MT PCR, and 100% apparent prevalence was recorded for cattle from the Auckland, East Cape and Northland regions, and 98.9% (86/87) in Waikato region (Table 1). In Group 2 cattle, there was a low prevalence (2.3%; 2/88) of genotypes *buffeli*, *chitose* and *type* 5 of *T. orientalis*; genotype *ikeda* was absent (Table 1). All four genotypes (i.e., *buffeli*, *chitose*, *ikeda* and *type* 5) were detected in all four geographical regions of the North Island, and *buffeli* had the highest prevalence (98.0%; 150/153), followed by *ikeda* (87.6%; 134/153), *chitose* (52.9%; 81/153) and *type* 5 (19.0%; 29/153). Herds in Auckland, East Cape and Waikato had the highest prevalence of genotype *buffeli* while those in Northland had both genotypes *ikeda* and *buffeli* in higher prevalences. In individual cattle, multiple genotypes of *T. orientalis* were more commonly detected than single genotypes (Figure 2). Mixed infections with genotypes *buffeli* and *ikeda* showed the highest prevalence (46.4%) followed by a combination of genotypes *buffeli*, *chitose* and *ikeda* (25.5%) (Figure 2).

Although genotype *buffeli* was prevalent in almost all farms in North Island, the relative average intensity of infection with genotype *ikeda* was dominant (with an average of 349,271 DNA copies), followed by *buffeli* (170,786), *chitose* (70,684) and *type* 5 (11,252) (Table 1). With the exception of East Cape, outbreaks in all other regions of North Island showed higher infection intensity with genotype *ikeda* than with genotype *buffeli* (Table 1).

Based on HCT values (130 blood samples), cattle in Group 1 were categorized as severely anaemic (HCT < 0.15; *n* = 49), mildly anaemic (HCT 0.15-0.24; *n* = 31) or non-anaemic/ normal (HCT > 0.24; *n* = 50) (Table 2). Haematocrit values were not available for the cattle in Group 2, but were assumed to be normal given that blood had been collected from clinically normal cattle and there was no indication of *ikeda* or its vector being present in the region. The apparent prevalences of genotypes *buffeli* and *ikeda* were high (100%) in severely anaemic cattle. However, in both mildly anaemic and non-anaemic cattle, the apparent prevalence of genotype *buffeli* was higher (96.8% and 92%, respectively). In non-anaemic cattle, the prevalence of genotype *ikeda* was lower (66%) than that of genotypes *buffeli* or *chitose* (Table 2).

**Table 2:** **Prevalence and intensities of** ***Theileria orientalis*** **genotypes detected using MT PCR in three anaemic categories**

**Category (** ***n*** **)** ^**a**^	**Apparent prevalence of genotype % (proportion)**				**Mean intensity of infection (mean no. of DNA copies)**			
	***ikeda***	***chitose***	***buffeli***	***type*** **5**	***ikeda***	***chitose***	***buffeli***	***type*** **5**
Severe anaemia^b^ (49)	100 (49/49)	34.7 (17/49)	100 (49/49)	8.2 (4/49)	445,347	68,915	157,443	88
Mild anaemia^c^ (31)	93.5(29/31)	38.7 (12/31)	96.8 (30/31)	16.1 (5/31)	429,380	167,705	260,844	9,518
No anaemia^d^ (50)	66 (33/50)	70 (35/50)	92 (46/50)	26 (13/50)	240,359	44,838	134,136	1,558

^a^ *n*, number of animals.

^b^Severe anaemia was defined if haematocrit value was < 0.15.

^c^Mild anaemia was defined if haematocrit value was 0.15-0.24.

^d^No anaemia was defined if haematocrit value was > 0.24.

(Haematocrit data were available for only 130 individuals of the tested 154 cattle from North Island).

Overall the intensity of infection with genotype *ikeda* was higher in infected cattle in severely anaemic, mildly anaemic and non-anaemic categories (Table 2). Intensity of infection with genotype *ikeda* was significantly higher (*P* = 0.009) in severely anaemic cattle than that in non-anaemic cattle (Table 3). In mildly anaemic cattle, the intensity of infection with genotype *type* 5 was significantly higher (*P* = 0.006) than in non-anaemic cattle (Table 3). Paired-sample *t*-test of data for severely anaemic cattle showed that intensity of infection with genotype *ikeda* was significantly higher than that with *buffeli* (*P* < 0.001) (Table 4).

**Table 3:** **Comparisons of intensities of four genotypes of** ***Theileria orientalis*** **among three anaemic categories**

**Genotype**	**Groups (** ***n*** **)** ^**a**^	**Intensity of infection (log DNA copy nos.)**		
		**Mean difference ± SE** ^**b**^	**95% CI** ^**c**^	***P*** **-value**
*ikeda*	Severe anaemia^d^ (49)	0.510 ± 0.20	0.129, 0.892	0.009
	Mild anaemia^e^ (29)	0.323 ± 0.22	-0.109, 0.755	0.143
	No anaemia^f^ (33)	Reference group		
*chitose*	Severe anaemia^d^ (17)	0.005 ± 0.28	-0.546, 0.556	0.986
	Mild anaemia^e^ (12)	0.238 ± 0.32	-0.386, 0.861	0.455
	No anaemia^f^ (35)	Reference group		
*buffeli*	Severe anaemia^d^ (49)	0.120 ± 0.14	-0.153, 0.393	0.390
	Mild anaemia^e^ (30)	0.199 ± 0.16	-0.113, 0.511	0.212
	No anaemia^f^ (46)	Reference group		
*type* 5	Severe anaemia^d^ (4)	-0.066 ± 0.58	-1.193, 1.061	0.909
	Mild anaemia^e^ (5)	1.442 ± 0.53	0.405, 2.479	0.006
	No anaemia^f^ (13)	Reference group		

^a^ *n*, number of animals.

^b^SE, standard error.

^c^CI, confidence interval.

^d^Severe anaemia was defined if haematocrit value was < 0.15.

^e^Mild anaemia was defined if haematocrit value was 0.15-0.24.

^f^No anaemia was defined if haematocrit value was > 0.24.

**Table 4:** **Comparisons of intensities of genotypes** ***ikeda*** **and** ***buffeli*** **within each anaemic category**

**Categories (** ***n*** **)** ^**a**^	**Genotypes**	**Intensity of infection (DNA copy nos.)**			
		**Median (min, max)** ^**b**^	**Mean**	**Differences of log mean (95% CI)** ^**c**^	***P*** **-value**
Severe anaemia^d^ (49)	*ikeda*	252,860	445,347	0.403 (0.34, 0.47)	< 0.001
		(247, 2242,034)			
	*buffeli*	108,368	157,443	Reference group	
		(680, 901,132)			
Mild anaemia^e^ (29)	*ikeda*	237,140	429,380	0.112 (-0.16, 0.39)	0.408
		(1,925, 2718,117)			
	*buffeli*	95,313	269,257	Reference group	
		(2,456, 3,698,674)			
No anaemia^f^ (33)	*ikeda*	74,688	240,359	-0.076 (-0.32, 0.17)	0.532
		(387, 3090,431)			
	*buffeli*	78,619	141,731	Reference group	
		(1,212, 1164,903)			

^a^ *n*, number of animals.

^b^min, minimum; max, maximum.

^c^CI, confidence interval.

^d^Severe anaemia was defined if haematocrit value was < 0.15.

^e^Mild anaemia was defined if haematocrit value was 0.15-0.24.

^f^No anaemia was defined if haematocrit value was > 0.24.

### SSCP-coupled sequencing of MT PCR amplicons

SSCP analysis of 50 of all 154 amplicons representing the genotypes *buffeli* (*n* = 15), *chitose* (*n* = 10), *ikeda* (*n* = 15) and *type* 5 (*n* = 10) showed four main profiles. Minor profile variation was detected within genotype *buffeli* (data not shown), which was reflected in a minute peak melting temperature difference (0.8-0.9°C). DNA sequencing of amplicons revealed that a nucleotide variation of 1.4% was linked to the target region (data not shown).

### Apparent prevalence of *T. orientalis* genotypes by *T. orientalis* PCR-HRM analysis and Ikeda TaqMan® qPCR

In Group 1, the prevalence of infection with all genotypes of *T. orientalis* by PCR-HRM was estimated at 99.4% (153/154) (Table 1), and the C*t* value ranged from 16.2 to 36.5 (mean 25.3 ± 4.2). Results for the TaqMan® qPCR revealed that 87.7% (135/154) of cattle in Group 1 were positive for genotype *ikeda* (Table 1), with C*t* values ranging from 20.9 to 35.9 (mean 27.2 ± 2.7). In Group 2, only two cattle (2.3%) were test-positive with genotype *buffeli* using PCR-HRM (Table 1).

### Comparison of performance of the three molecular diagnostic methods

All samples that were test-positive by PCR-HRM were also test-positive by MT PCR assay for one or more genotypes of *T. orientalis*. All samples test-negative by PCR-HRM were also test-negative for *T. orientalis* genotypes *buffeli*, *chitose*, *ikeda* or *type* 5 by MT PCR. Of the 135 samples that were test-positive by Ikeda TaqMan® qPCR, 134 were test-positive for genotype *ikeda* in the MT PCR assay. The diagnostic specificity of the MT PCR detecting genotype *ikeda* (98.9%; 95% PI: 96.4, 99.8%) was calculated to be comparable to that of the Ikeda TaqMan® qPCR (97.4%; 95% PI: 95.1, 98.8%); the diagnostic sensitivity of the MT PCR (98.0%; 95% PI: 94.6, 99.6%) was also comparable to that of Ikeda TaqMan® qPCR (97.1%; 95% PI: 94.2, 98.8%) using a cut-off for test positivity of ≥ 1 DNA copies (Table 5). The diagnostic specificity (97.8%; 95% PI: 94.5, 99.3%) and sensitivity (98.9%; 95% PI: 97.0, 99.8%) of the MT PCR for the simultaneous detection of genotypes *buffeli*, *chitose*, *ikeda* and *type* 5 of *T. orientalis* were also very comparable with values calculated for PCR-HRM (96.5%; 95% PI: 94.3, 98.3%; 97.6%; 95% PI: 95.6, 99.0%, respectively) (Table 6). Overall, there was an excellent agreement (posterior median PABAK > 0.867 for all iterations) in results between the MT PCR and PCR-HRM assays using samples from both groups of cattle.

**Table 5:** **Diagnostic test performance of MT PCR and Ikeda TaqMan® qPCR**

**Outcome**	**Median (95% PI)** ^**a**^
**MT PCR**	
Diagnostic sensitivity (%)	98.0 (94.6, 99.6)
Diagnostic specificity (%)	98.9 (96.4, 99.8)
**Ikeda TaqMan® qPCR**	
Diagnostic sensitivity (%)	97.1 (94.2, 98.8)
Diagnostic specificity (%)	97.4 (95.1, 98.8)
**Estimated true prevalence**	
Group 1 (%)	88.1 (82.5, 93.1)
Group 2 (%)	0 (0, 0.12)
**Agreement between tests**	
PABAK in group 1	94.0 (89.0, 97.2)
PABAK in group 2	94.1 (88.0, 97.7)
**Conditional dependence between tests** ^**b**^	
*ρ+*	0.371 (0.004, 0.764)
*ρ-*	0.226 (-0.006, 0.674)
**Model fit**	
pD	7.6
DIC	28.4

The diagnostic sensitivity of the MT PCR was defined based on a cut-off of ≥ 1 DNA copies.

^a^PI, probability interval.

^b^ *ρ+* is the conditional correlation between MT and Ikeda TaqMan® qPCRs for infected animals, and *ρ-* is the conditional correlation for uninfected animals.

PABAK = prevalence-adjusted bias-adjusted Cohen’s kappa (*ĸ*) statistic.

pD = effective dimension (model complexity).

DIC = Deviance information criterion, a generalisation of Akaike’s Information Criterion (AIC).

**Table 6:** **Diagnostic test performance of MT PCR and HRM analysis**

**Outcome**	**Median (95% PI)** ^**a**^
**MT PCR**	
Diagnostic sensitivity (%)	98.9 (97.0, 99.8)
Diagnostic specificity (%)	97.8 (94.5, 99.3)
**PCR-HRM analysis**	
Diagnostic sensitivity (%)	97.6 (95.6, 99.0)
Diagnostic specificity (%)	96.5 (94.3, 98.3)
**Estimated true prevalence**	
Group 1 (%)	98.2 (95.5, 99.6)
Group 2 (%)	0 (0, 2.14)
**Agreement between tests**	
PABAK in group 1	95.2 (90.2, 98.0)
PABAK in group 2	94.0 (87.3, 97.9)
**Conditional dependence between tests** ^**b**^	
*ρ+*	0.301 (0.006, 0.715)
*ρ-*	0.480 (0.106, 0.772)
**Model fit**	
pD	12.8
DIC	35.1

The diagnostic sensitivity of the MT PCR was defined based on a cut-off of ≥ 1 DNA copies.

^a^PI, probability interval.

^b^ *ρ+* is the conditional correlation between MT PCR and PCR-HRM outcomes for infected animals, and *ρ-* is the conditional correlation for uninfected animals.

PABAK = prevalence-adjusted bias-adjusted Cohen’s kappa (*ĸ*) statistic.

pD = effective dimension (model complexity).

DIC = Deviance information criterion, a generalisation of Akaike’s Information Criterion (AIC).

## Discussion

This is the first application of the newly developed semi-quantitative MT PCR assay [14] for the simultaneous detection and differentiation of four genotypes of *T. orientalis* in New Zealand. The MT PCR assay uses three independent markers (i.e., genotype-specific primers) in the *p23* gene, *MPSP* gene and ITS-1 of nuclear ribosomal DNA regions for the simultaneous detection of genotypes *buffeli, chitose* and *type* 5, and *ikeda*, respectively. In contrast, the TaqMan® qPCR and PCR-HRM assays (D. J. Pulford, and A. M. J. McFadden, unpublished observations) both employ primers to the *MPSP* gene to detect genotypes *ikeda* and *T. orientalis*, respectively. Comparison of the MT PCR with the two other PCR techniques using Bayesian latent class modeling showed that the diagnostic sensitivities (≥ 97.1%) and specificities (≥ 96.5%) of the three molecular tools were comparable.

Using both MT PCR and PCR-HRM, we observed that 99.4% (153/154) and 2.3% (2/88) of cattle were infected with *T. orientalis* using samples from the North (Group 1) and South (Group 2) Islands of New Zealand, respectively. Using the MT PCR assay, we identified all four genotypes of *T. orientalis* (i.e., *buffeli*, *chitose*, *ikeda* and *type* 5) in all four regions (i.e., Auckland, East Cape, Northland and Waikato) studied on the North Island, with *buffeli* having the highest apparent prevalence (98.0%). Using this assay, we were also able to detect the presence of genotypes *chitose* and *type* 5 on the South Island, not detectable by the other two tests, and to confirm the absence of genotype *ikeda*, in accordance with results for both MT PCR and Ikeda TaqMan® qPCR.

In 1982, James et al. [13] reported *T. orientalis* cases in New Zealand from both dairy and beef cattle, mostly from Wellsford (the northern-most town in the Auckland region). The detection of *T. orientalis* in Southland cattle in the present study suggests that there may have been some recent tick activity in the region, although the information on the distribution of the proposed tick-vector (*Haemaphysalis longicornis*) of *T. orientalis* in New Zealand is lacking for the South Island [20]. A possible reason for the detection of *T. orientalis* in the Southland might have been the transportation of cattle from the North to the South Island, although this explanation seems unlikely, as beef cattle from Southland tested here were less than two years of age, and because the movement of young beef cattle from the North to the southern South Island for fattening is uncommon in New Zealand. This proposal and the detection of *T. orientalis* in the South Island [21] prompt a future investigation into the current distribution of *H. longicornis* on the South Island of New Zealand.

This study reports the first detection of genotype *ikeda* in New Zealand using ITS-1. Previously, McFadden et al. [5] reported genotype *chitose* in New Zealand using primers targeting the mitochondrial gene *cox*3 and a region in the nuclear ribosomal small subunit (18S). Although previous studies have identified all four genotypes (i.e., *buffeli*, *chitose*, *ikeda* and *type* 5) using conventional PCR, employing primers to the *p23* and *MPSP* genes (D. J. Pulford, and A. M. J. McFadden, unpublished observations), the apparent prevalence of *T. orientalis* determined here is inferred to be considerably more accurate than conventional PCR, due to a 1,000 times higher analytical sensitivity of the MT PCR [14]. In addition, the MT PCR assay is able to estimate the infection intensity for each genotype based on the DNA copy number detected. These results reveal a relationship between a high intensity of genotype *ikeda* and severe anaemia in cattle affected by oriental theileriosis based on HCT values (< 0.15), suggesting that genotype *ikeda* is the pathogenic genotype in New Zealand, as is the case for regions in Australia [14,15]. The apparent absence of genotype *ikeda* from cattle from Southland, where no outbreaks have been reported, also provide support for this proposal. We also found that the intensity of infection with genotype *type* 5 was significantly higher (*P* = 0.006) in mildly anaemic cattle than in non-anaemic cattle (Table 3); however, this result should be interpreted with caution due to small sample size in each category of cattle tested here.

It appears that genotype *ikeda* is the main pathogenic genotype involved in oriental theileriosis in cattle in New Zealand. Although both genotypes *chitose* and/or *ikeda* were suggested to be linked to symptomatic oriental theileriosis in cattle in Australasia, based on the presence and/or prevalence of genotypes by conventional molecular diagnostic tools [3-7,10-12], the application of MT PCR in Australia to detect and estimate the infection intensities of individual *T. orientalis* genotypes provided evidence that genotype *ikeda* was the main pathogenic genotype present in south-eastern Australia [15]. The present study shows a similar finding for New Zealand. However, based on conventional molecular tools, McFadden et al. [5] showed that genotype *chitose* was associated with a varying severity of anaemia in dairy cattle infected with *T. orientalis*. Subsequently, in outbreaks of bovine theileriosis in New Zealand in 2012 and 2013, Pulford et al. (D. J. Pulford, and A. M. J. McFadden, unpublished observations) have reported, for the first time, that genotype *ikeda* was linked to clinical oriental theileriosis. Furthermore, Gias et al. (E. Gias, D. J. Pulford, and A. M. J. McFadden, unpublished observations) also found a relationship between higher infection intensity of the genotype *ikeda* and anaemia.

The present study showed that MT PCR and PCR-HRM had very similar diagnostic specificities and sensitivities for the detection of genotypes *buffeli*, *chitose*, *ikeda* and *type* 5 of *T. orientalis* (Tables 5 and 6). In addition, MT and Ikeda TaqMan® qPCRs had similar diagnostic specificity and sensitivity for detecting genotype *ikeda*, and the results from MT PCR were comparable to the combined results obtained by both PCR-HRM and Ikeda TaqMan® qPCR (which are the molecular tools currently used in New Zealand; D. J. Pulford, and A. M. J. McFadden, unpublished observations). The estimated cost per sample (including the cost for DNA extraction) by MT PCR is AUD 22, which is approximately twice that of PCR-HRM and Ikeda TaqMan® qPCR (i.e., AUD 10). MT PCR assay used here can test 12 samples per hour, whereas the time taken to test 12 samples by PCR-HRM is 1.5 h and an additional 1.5 h for Ikeda TaqMan® qPCR (i.e., a total of 3 hours). Importantly, MT PCR assay is performed in a robotic platform reducing the labour, contamination and PCR inhibition involved with many conventional and qPCR assays. In addition, the analytical MT PCR assay allows a qualitative (detection and differentiation) and semi-quantitative evaluation of four distinct genotypes of *T. orientalis* at once, which cannot be achieved using the other two PCRs.

## Conclusion

In conclusion, MT PCR assay used here has some important advantages over other diagnostic techniques currently used within and outside of Australasia for the detection and differentiation of *T. orientalis.* Findings from the present study provide further support for the proposal that MT PCR assay could be used to predict the risk of clinical disease developing in cattle, based on DNA copy number thresholds of pathogenic and apathogenic genotypes. Although PCR-HRM and Ikeda TaqMan® qPCR have proven to be useful diagnostic tools in a recent epidemic of oriental theileriosis in New Zealand, MT PCR allows the simultaneous detection of four common genotypes (*buffeli*, *chitose*, *ikeda* and *type* 5) of *T. orientalis* complex and an estimation of the intensity of infection with these genotypes, thereby making it a useful tool for in-depth epidemiological and transmission studies.

## Abbreviations

ITS-1: First internal transcribed spacer
HCT: Haematocrit
*MPSP*: Major piroplasm surface protein
MT PCR: Multiplexed tandem PCR
PABAK: Prevalence-adjusted bias-adjusted Kappa
PCR-HRM: PCR-high resolution melting
*p23*: 23-kDa piroplasm surface protein
PI: Probability interval
SSCP: Single-strand conformation polymorphism
